# Significance of Thymosin β4 and Implication of PINCH-1-ILK-α-Parvin (PIP) Complex in Human Dilated Cardiomyopathy

**DOI:** 10.1371/journal.pone.0020184

**Published:** 2011-05-19

**Authors:** Nikolai Sopko, Yilu Qin, Amanda Finan, Alisher Dadabayev, Sravanthi Chigurupati, Jun Qin, Marc S. Penn, Sudhiranjan Gupta

**Affiliations:** 1 Division of Cardiology, Department of Internal Medicine, Cardiovascular Research Institute, Texas A & M Health Science Center, College of Medicine, Scott and White, Central Texas Veterans Health Care System, Temple, Texas, United States of America; 2 Department of Stem Cell and Regenerative Medicine, Lerner Research Institute, Cleveland Clinic, Cleveland, Ohio, United States of America; 3 Department of Molecular Cardiology, Lerner Research Institute, Cleveland Clinic, Cleveland, Ohio, United States of America; University of Bristol, United Kingdom

## Abstract

Myocardial remodeling is a major contributor in the development of heart failure (HF) after myocardial infarction (MI). Integrin-linked kinase (ILK), LIM-only adaptor PINCH-1, and α-parvin are essential components of focal adhesions (FAs), which are highly expressed in the heart. ILK binds tightly to PINCH-1 and α-parvin, which regulates FA assembly and promotes cell survival via the activation of the kinase Akt. Mice lacking ILK, PINCH or α-parvin have been shown to develop severe defects in the heart, suggesting that these proteins play a critical role in heart function. Utilizing failing human heart tissues (dilated cardiomyopathy, DCM), we found a 2.27-fold (p<0.001) enhanced expression of PINCH, 4 fold for α-parvin, and 10.5 fold (p<0.001) for ILK as compared to non-failing (NF) counterparts. No significant enhancements were found for the PINCH isoform PINCH-2 and parvin isoform β-parvin. Using a co-immunoprecipitation method, we also found that the PINCH-1-ILK-α-parvin (PIP) complex and Akt activation were significantly up-regulated. These observations were further corroborated with the mouse myocardial infarction (MI) and transaortic constriction (TAC) model. Thymosin beta4 (Tβ4), an effective cell penetrating peptide for treating MI, was found to further enhance the level of PIP components and Akt activation, while substantially suppressing NF-κB activation and collagen expression—the hallmarks of cardiac fibrosis. In the presence of an Akt inhibitor, wortmannin, we show that Tβ4 had a decreased effect in protecting the heart from MI. These data suggest that the PIP complex and activation of Akt play critical roles in HF development. Tβ4 treatment likely improves cardiac function by enhancing PIP mediated Akt activation and suppressing NF-κB activation and collagen-mediated fibrosis. These data provide significant insight into the role of the PIP-Akt pathway and its regulation by Tβ4 treatment in post-MI.

## Introduction

The integrin-linked kinase (ILK) is a major focal adhesion protein which links integrin's to actin and maintains a cell to extracellular matrix interaction for diverse physiological processes including embryonic development, growth, cellular proliferation, differentiation and survival [1], [2]. Widely expressed in various cell types and tissues, ILK is known to form a tripartite complex with LIM-only protein PINCH and Parvin. Originally thought as a kinase [3], recent structural and genetic studies have indicated that ILK is a pseudo-kinase which functions as a scaffold to bind various proteins including the integrin β cytoplasmic tail, and the cytoskeletal adaptors PINCH and parvin [4], [5]. The high affinity interactions of PINCH-1-ILK-α-Parvin (PIP) are particularly important because they have been found in many cell types and are essential for focal adhesions that link extracellular matrix and cytoskeleton. PINCH has two isoforms, PINCH-1 and PINCH-2. Parvin has three isoforms; α-, β-and γ-Parvin. ILK, PINCH-1 and α/β-parvin are highly abundant in the heart and play an important role in cardiac function as suggested by a growing amount of evidences [2], [6], [7]. Notably, conditional ILK deletion in the mouse heart causes spontaneous dilated cardiomyopathy (DCM) and sudden death at 6 to 12 weeks of age [8], [9]. Cre expression driven by the muscle creatine kinase promoter also resulted in efficient deletion of ILK from skeletal muscle. Surprisingly, these mice showed no obvious muscle defects and grew normally, although early death from DCM precluded development of later defects in ilk^−/−^ skeletal muscle. The DCM phenotype in *ilk* ^−/−^ hearts is similar to mice with cardiac-specific ablation of ß_1_ integrin [10] and focal adhesion kinase (FAK) [11]. However, loss of ILK causes early spontaneous onset of DCM, whereas ß_1_ integrin and FAK ablation causes DCM in response to an applied cardiac stress, suggesting ILK is an effector of other critical signaling pathways beyond FA. In this regard, the serine/threonine kinase Akt Ser473 phosphorylation was suppressed in *ilk* ^−/−^ [8] and PINCH1/2^−/−^ [12]. The phosphorylation of Ser 473 of Akt activates the kinase, which in turn transduces antiapoptotic “survival” signals in a number of cells, including cardiomyocytes [13], [14]. Given the data that PIP is critically involved in regulating Akt Ser-473 phosphorylation [8], PIP and its pathway to Akt activation are likely involved in mediating heart disease progression. It is postulated that the activation of Akt and the consequent inhibition of GSK-3 may protect cells from apoptosis. Therefore, Akt activation in failing DCM hearts could be a protective antiapoptotic mechanism. Since, animal-based studies have recently indicated that Akt/PKB is critically regulated by ILK during cardiac function and dysfunction [8], [15], [16] and that Akt activation is dependent on the ILK/PINCH interaction [17], we hypothesized that the phosphorylation of Akt Ser 473 might also be elevated in DCM hearts. Interestingly, a study on myocardial infarction in mice showed that thymosin β4 (Tβ4), a monomeric G-actin protein, targets ILK-PINCH-1 leading to the activation of Akt for cardiac protection after ischemic insult [8], [15], [16]. A follow up study showed that Tβ4 induces adult epicardial progenitor cells mobilization and neovascularization following cardiac injury [18]. However, the mechanistic relationship between Tβ4, ILK-PINCH-1 and cardiac protection still remain undefined. Moreover, the complete profiling of ILK and its binding partners, like PINCH-1 and Parvin are still not known in the failing human heart (DCM) or in experimentally induced MI or pressure overload (transaortic constriction, TAC) models. Herein, we report the activation cascade of ILK, PINCH and Parvin complexes in failing human hearts (DCM), and in murine TAC and in MI models. Our data provide the first insight into the role of PIP in human DCM in correlation with mouse MI or TAC model. We also show that Tβ4 treatment may target the PIP-Akt pathway and reduce inflammatory mediators, thereby, providing cardiac protection in the MI model. Importantly, we showed that Tβ4 limits the cardiac protection in an MI model in the presence of wortmannin, an Akt inhibitors suggesting the critical role of Akt in relation to Tβ4 during cardiac protection.

## Results

### Expression of PIP in DCM human hearts

**Figure 1:**
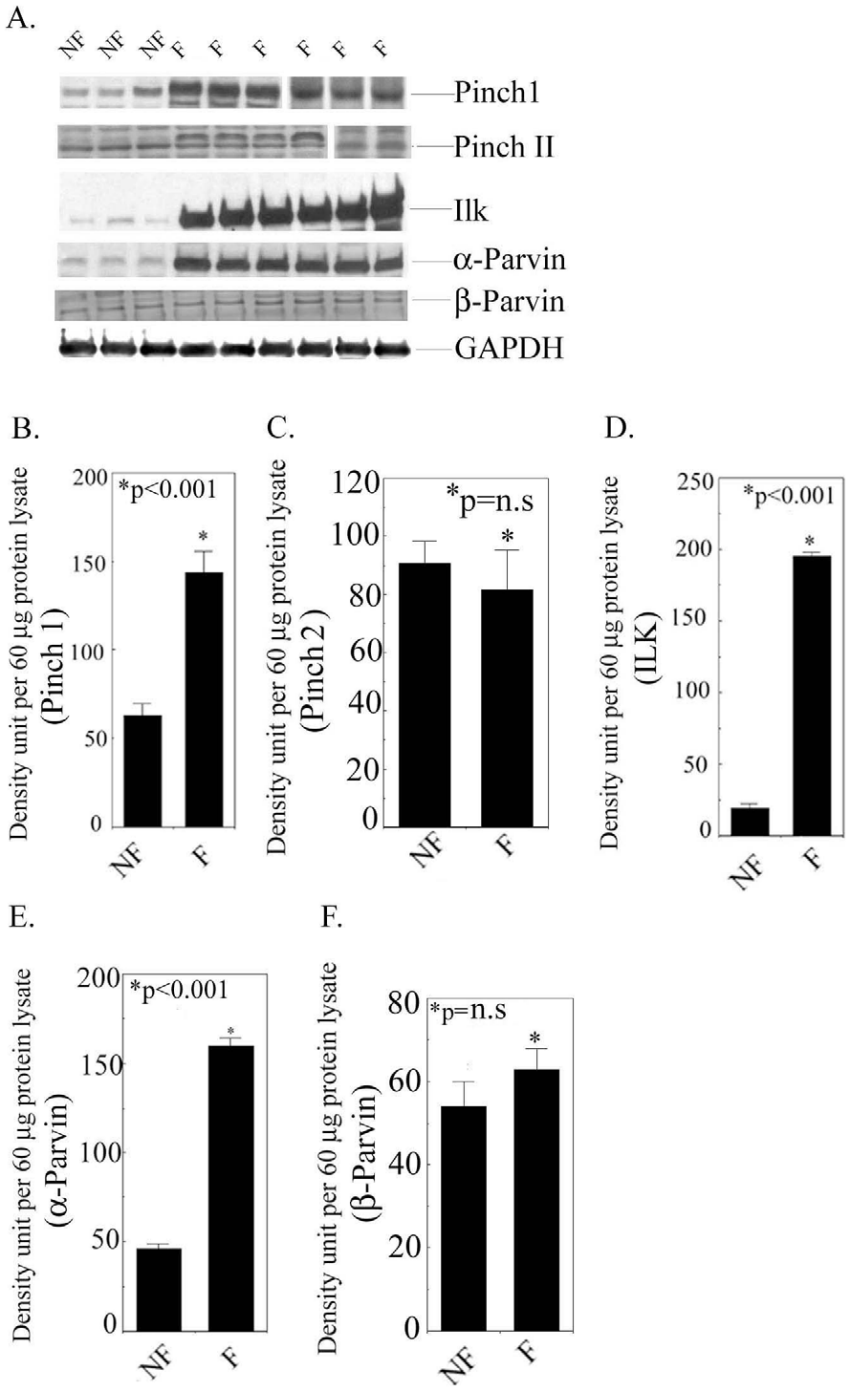
ILK and its associated protein levels in F and NF hearts. A Expression of ILK, PINCH-1, PINCH-II, α-Parvin, β-Parvin, and ILK in NF and F human left ventricular lysates. GAPDH was the loading control. Six NF hearts and nine F hearts were used for all proteins except for PINCH-1 (12 F hearts). Shown here are representative examples of these samples. Note that two representative gels from two different runs were shown for PINCH-1 and PINCH-II respectively. Also, in some of the F hearts, there exists a higher molecular weight band above PINCH-II. The origin of the band is not clear. B–F Quantitation of protein expression in the F and NF hearts. (B) PINCH-1, (C) PINCH-II, (D) ILK), (E) α-Parvin, and (F) β-Parvin. *p<0.001 F *vs.* NF (paired t-test), n = 6 for NF hearts and N = 9 for DCM hearts for PINCH-1 ILK and α-Parvin protein expression. p = n.s for PINCHII and β-Parvin protein.

To determine the role of PIP in human DCM, we first examined the ILK and PINCH-1 levels in non-failing (NF) and DCM or failing (F) human hearts. NF hearts from six individual donors and F hearts from nine DCM patients were used for the analysis. Our data showed a 2.27-fold enhanced expression of PINCH-1 and 10.5 fold increased expression of ILK in F human hearts in comparison to NF human hearts (p<0.001) (Fig. 1). An actin-binding protein α-Parvin, crucial for connecting the ILK/PINCH-1 complex to the actin cytoskeleton [19], was also found to be elevated in the F hearts (Figure 1 E). Additionally, we determined the expression levels of PINCH-II and β-Parvin, which did not show any significant changes in DCM hearts (Fig. 1 C and F).

### Status of ILK and PINCH-1 complexes and AKT in F and NF human hearts

**Figure 2:**
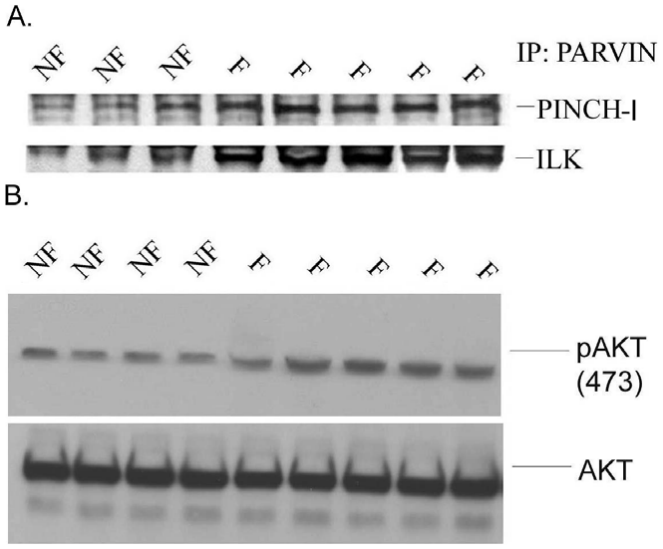
The interaction of ILK/PINCH-1 in failing (F) human hearts. A Immunoprecipitation of the α-Parvin/ILK/PINCH-1 complex in the F and NF human hearts. Extracts were immuno-precipitated with anti-PARVA antibody 1D4 (α-P). Immuno-precipitated proteins were immunoblotted with monoclonal anti-PARVA clone 3B5 antibody or with monoclonal anti-ILK antibody and monoclonal anti-PINCH antibody (Cell signaling, Inc). B. Phosphorylation of Akt Ser473 in representative F and NF hearts. The total Akt level is shown below. Six NF hearts and nine F hearts were used for all proteins except for PINCH-1 (12 F hearts). Shown here are representative examples of these samples. Results are presented as five different mice (n = 5).

In order to determine the level of the ILK and PINCH complex separately during heart failure, we performed co-immunoprecipitation studies. The experiments determined the levels of the ILK/PINCH-1 complex in both F and NF hearts. Based on the fact that N-terminal ILK ARD binds to PINCH-1 whereas C-terminal ILK kinase domain binds to α-Parvin and the three proteins form a ternary complex in cells [19], we used α-Parvin antibody/α-Parvin to effectively pull down the ILK/PINCH-1 complex – an approach that was also previously reported to pull down ILK/PINCH-1 in cardiomyocytes [13], [14]. Consistent with Figure 1A, the association of ILK with PINCH-1 was also significantly elevated in the F human hearts (Figure 2). Our data showed that both PINCH and ILK (1.8- and1.4-fold respectively, p<0.001) complexes were significantly increased in all F hearts, compared to NF hearts (Fig. 2 A). Note that because we used α-Parvin antibody to pull down the ILK/PINCH complex, the data suggest that the ternary PINCH-ILK-α-Parvin complex (PIP) is also elevated.

Akt is activated as a downstream consequence of growth factor signaling through phosphoinositide-3 kinases, and activation of Akt has been associated with protection from apoptosis in cardiac myocytes [20]. Because progressive myocyte apoptosis may contribute to heart failure, we examined the activation status of Akt by western blotting with a phospho-specific (Ser 473) antibody (Fig. 2B). We observed a marked increase in Akt phosphorylation in F human hearts compared with NF hearts, but absolute protein levels were invariant.

### Expression of PIP and phosphorylation of Akt in transaortic constriction (TAC) and myocardial infarction model: Effect of Tβ4 in MI

**Figure 3:**
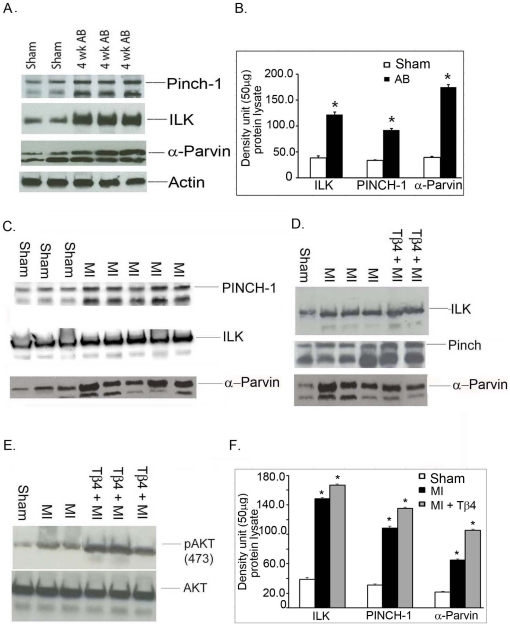
Expression profile for PIP in TAC and MI. A. Individual PIP proteins were analyzed by Western blotting in mice 4 weeks after aortic banding (AB) Upper panel shows the protein expression of Pinch-1, the middle panel shows ILK and the bottom panel shows α-Parvin. Actin is used as a loading control. B. Quantification of panel A is shown on right side of panel A. The error bars represent the average ± S.E. of five different mice repeated in an independent experiments (n = 5, *p<0.001 compared to sham group, student t-test). Expression profile of PIP in MI and the effect of Tβ4 on MI. C. Western blot analysis of ILK, Pinch-1 and α- Parvin after 7 days post-MI. D. Western blot analysis of ILK, Pinch-1 and α- Parvin in Tβ4 treated MI mice. E. Right panel shows western blotting analysis of Akt 473 phosphorylation as well as total AKT level in 7 days post-MI mice. F. Quantification of blots from 3C, 3D and 3E are shown on right side of panel of 3E. Results are presented in five different mice (n = 5,* p<0.01 compared to sham or MI group, student t-test). The one way ANOVA test among the groups of MI and MI+Tβ4 also showed significant at p<0.05 level.

To determine the expression of the PIP complex in the TAC model, we performed immuno-blot analysis using their specific antibodies. Our data showed a significant up regulation of both ILK, PINCH and α-Parvin protein after 4 weeks of TAC (Fig. 3 A and B). Compared to sham, TAC mice showed a 3.16 fold increase of ILK (38.41±3.29 *vs.* 121. 58±5.50, p<0.001), 2.79 fold for PINCH (32.84±1.75 *vs.* 91.73±2.93, p<0.001) and 4.43 fold increase in α-Parvin (39.48±1.57 *vs.* 174.94±4.97, p<0.001). These data suggest that the PIP complex is also elevated in the murine pressure overload model.

To determine the expression of ILK, PINCH-1 and α-Parvin after MI, we performed immuno-blot analysis using their specific antibodies. Our data showed a significant up regulation of ILK, PINCH and α-Parvin protein after 7d post-MI (Fig. 3 C and F). Compared to sham, MI mice showed a 3.82 fold increase of ILK (38.71±2.48 *vs.* 148.24±1.87, p<0.01), a 3.48 fold increase of PINCH-1 (31.20±1.03 *vs.* 108.73±2.51, p<0.001) and a 3.61 fold increase in α-Parvin (29.58±2.54 *vs.* 107.94±3.91, p<0.001). In order to assess the role of Tβ4 in regulating the PIP complex, Tβ4 was injected into the myocardium and we observed a further elevation of both ILK (148. 73±2.51 *vs.* 166.71±1.41) and PINCH-1 (108.73±2.51 *vs.* 135.24±1.73) proteins (Fig. 3 D and F) but no further elevation of α-Parvin. Additionally, we determined the phosphorylation of Akt (Ser473) in MI mice treated with Tβ4. The data are shown in Fig. 3E and F. We observed a 2.98 fold increase in Akt (Ser473) phosphorylation (21.75±0.69 *vs.* 65.02±1.48, p<0.001) in MI mice which increased an additional 1.61-fold after Tβ4 treatment (65.02±1.48 *vs.* 105.27±1.49) compared to non-treated MI mice.

### Effect of Tβ4 on cardiac function

**Figure 4:**
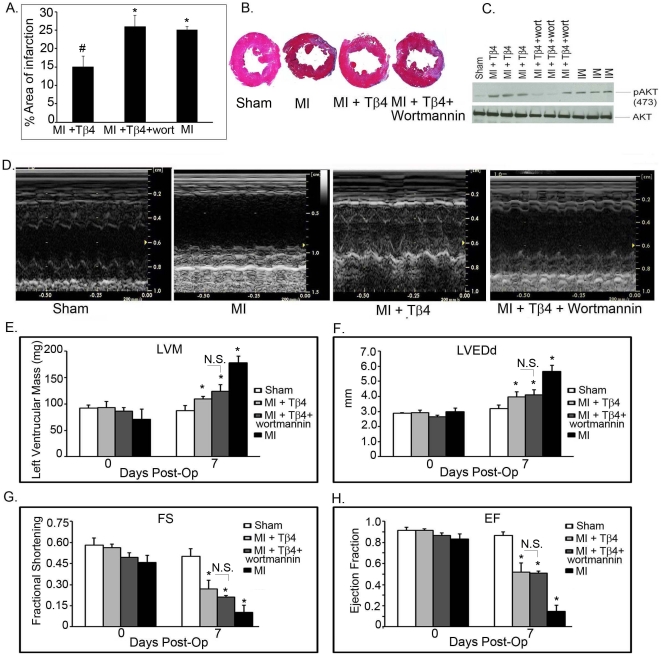
Infarct size and Echocardiographic analysis of Tβ4 and Tβ4+ wortmannin treated MI mice. A. The percent infarct size 7 days post-MI as determined by the epicardial infarct length divided by the epicardial left ventricle circumference in axial sections prepared with Masson's trichrome. The error bars represent the average ± S.E. of five different mice in an independent experiments (#p = 0.02 and was significant when compared MI *vs.* MI+Tβ4, *p = 0.69 and considered as non-significant in one way ANOVA analysis when compared to MI *vs.* MI+Tβ4+ wortmannin) B. Representative is the images of hearts after MI in the presence and absence of Tβ4 and wortmannin. C. Western blots analysis of phosphorylation of Akt Ser473 in of Sham (WT), MI, Tβ4 treated MI and MI+Tβ4+ wortmannin treated mice of 7 days post-MI. The total Akt level is shown below. D. The echocardiogram picture of Sham, MI, Tβ4 treated MI and MI+Tβ4+ wortmannin treated mice after 7 days of Tβ4 treatment. E–H Quantification of LVH, FSH, LVEDd and EF of the above mice after 7 days of MI. Results are presented in five different mice (n = 5, *p<0.001 compared to sham or MI group at 7 days post-op, student's t-test). The above parameters were not significant at day zero. The one way ANOVA test among the groups of MI and MI+Tβ4 also showed significant at p<0.05 level. The EF and FS in wortmannin +MI+Tβ4 treated group showed non- significant (N.S) when compared to MI+Tβ4 group.

Infarct size was evaluated in sham, MI, MI+Tβ4, and MI+Tβ4+ wortmannin groups 7 days post-MI (n = 5) by measuring the percent area of infarction in axial sections prepared with Masson's trichrome. Analysis demonstrated smaller infarct sizes in Tβ4 treated MI animals compared to MI only (15±3% *vs.* 25±3%, # p = 0.02). Furthermore, treatment with wortmannin in addition to Tβ4, abrogated the infarct attenuating effects of Tβ4 resulting in infarct areas comparable to MI (26±3% *vs.* 25±3%, *p = 0.69, Fig. 4A, 4B). Moreover, western blot analysis showed a significant inhibition of pAkt (Ser473) in MI+Tβ4+ wortmannin groups (Fig. 4C) indicating that wortmannin prevented Akt phosphorylation.

Additionally, we assessed cardiac function in sham, MI, MI+Tβ4, and MI+Tβ4+ wortmannin groups *via* transthoracic echocardiography before and 7 days after MI. There were no differences between groups prior to MI. Non-treated MI mice had the greatest decrease in cardiac function and increase in LV mass compared to sham operated mice (ejection fraction (EF), 0.14±0.06 *vs.* 0.87±0.03, p<0.01; end diastolic dimension (EDD), 5.6±0.4 mm *vs.* 3.2±0.2 mm, p<0.01; LV mass (LVM), 178.5±12.2 mg *vs.* 87.7±8.9 mg) (Fig. 4D–H). Mice receiving Tβ4 had a significantly improved cardiac function compared to untreated MI mice with Tβ4 only treatment having the greatest attenuating effect (EF, 0.52±0.08 *vs.* 0.14±0.06, p<0.01; EDD, 4.0±0.3 mm *vs.* 5.6±0.4 mm, p<0.05; LVM, 109.6±5.1 mg *vs.* 178.5±12.2 mg, p<0.05) (Fig. 4D, 4E–H).

Treatment with wortmannin moderately attenuated the beneficial effect of Tβ4 in MI model. Compared to Tβ4-treated mice, wortmannin treated mice showed a slight decrease in ejection fraction (0.52±0.08 *vs.* 0.50±0.02, p = 0.91) and fractional shortening (0.27±0.06 *vs.* 0.21±0.01, p = 0.36) as assessed by echocardiography (Fig. 4D, 4E–H).

### Effect of Tβ4 on NF-κB activation after MI

**Figure 5:**
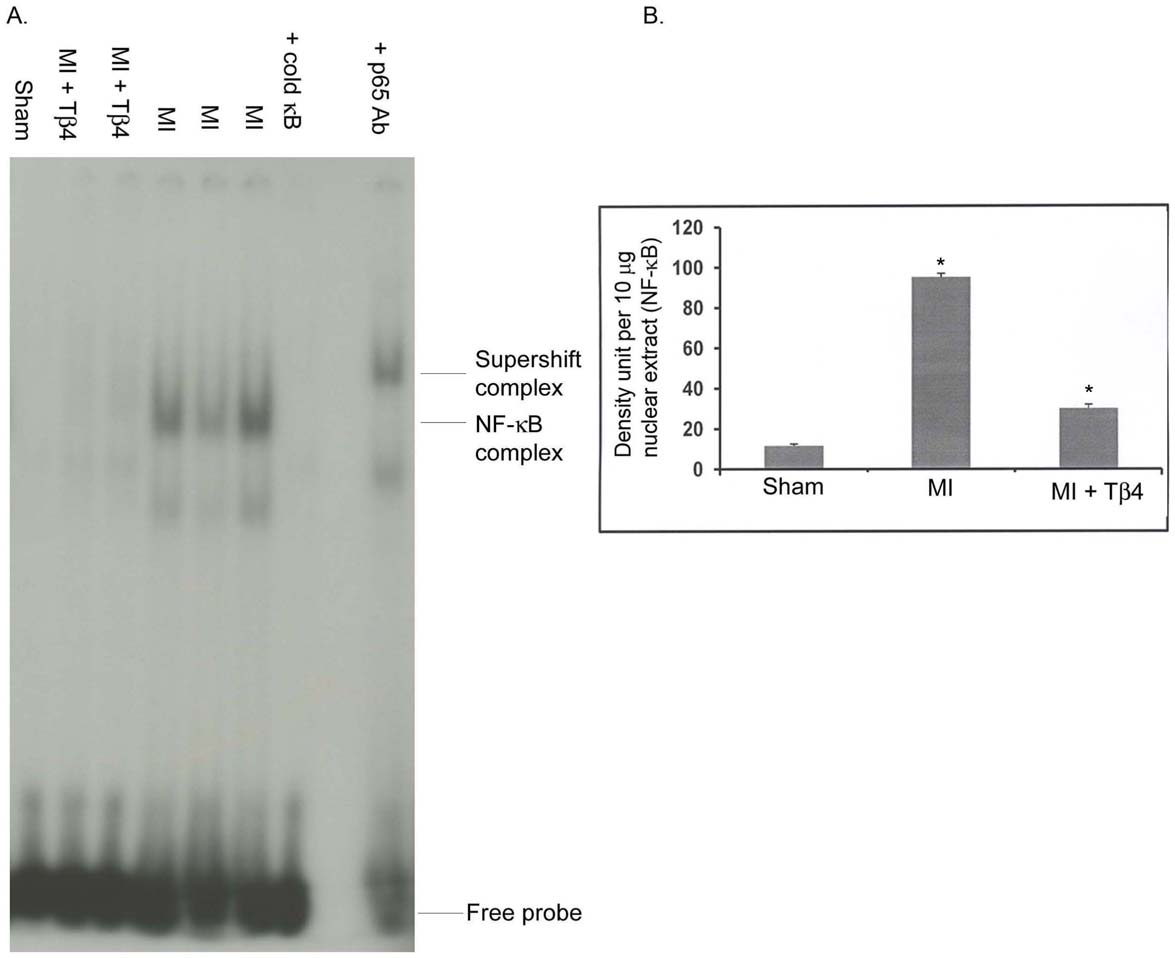
Tβ4 inhibits NF-κB translocation in MI mice. A. Mice were subjected to MI and Tβ4 was injected and mice were kept for 7 days. NF-κB activation was measured by gel mobility shift assay using 32p NF-κB DNA as a probe. Cold NF-κB DNA was used for competition analysis. A p65 antibody was used for super shift analysis. B. Semi-quantification of panel A is shown on right side of panel A. Results are presented as five different mice (n = 5, *p<0.001 compared to sham or MI group, student's t-test). The one way ANOVA test among the groups of MI and MI+Tβ4 also showed significant at p<0.05 level.

To find out the role of Tβ4 in inflammation, we analyzed NF-κB activation in 7d post-MI in mice treated with Tβ4. An 8.14 fold NF-κB activation (11.7±0.84 *vs.* 95.25±1.72, p<0.001) was observed in 7d post-MI. Our data further indicate that Tβ4 significantly reduces NF-κB activity in post-MI mice compared to MI mice (95.25±1.72 *vs.* 30.10±1.91, p<0.001) (Fig. 5).

### Effect of Tβ4 on collagen expression after MI

**Figure 6:**
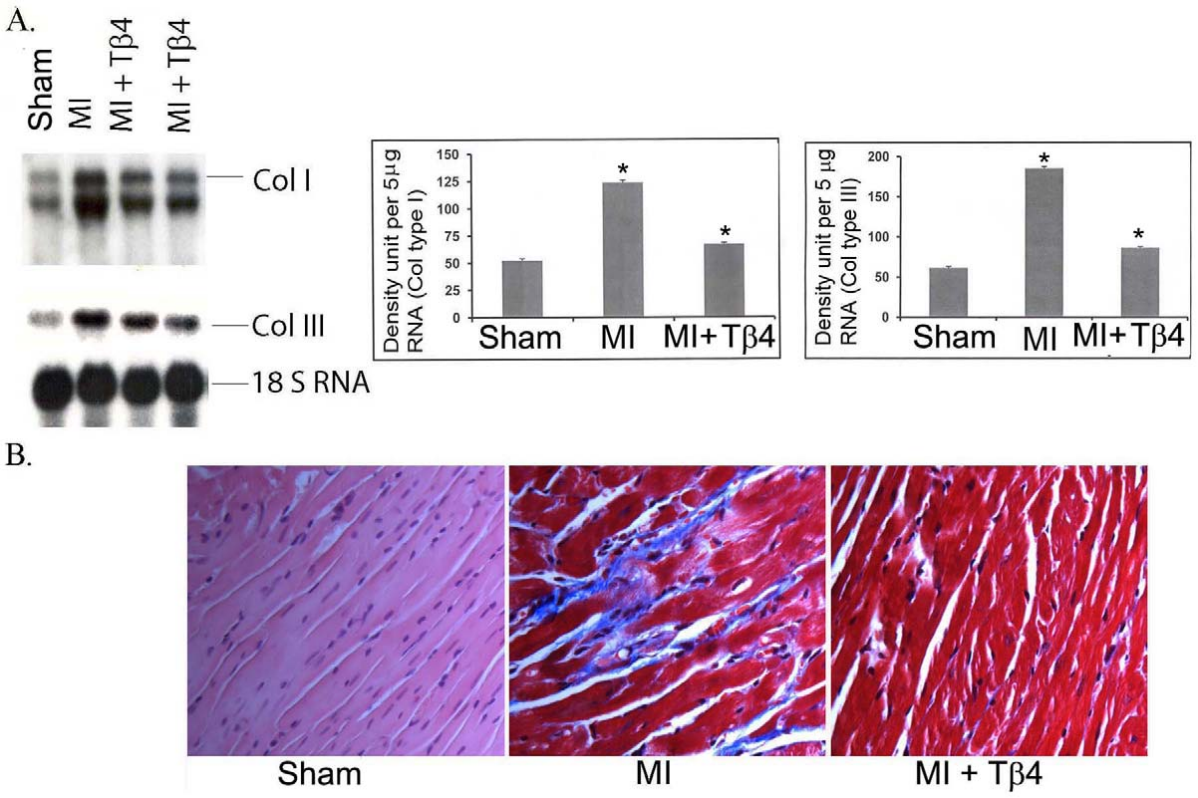
Tβ4 attenuates cardiac fibrosis. A. Collagen type I and type III mRNA Expression was performed using their respective cDNA probes. Quantification of panel A is shown on right side of panel A. Results are presented as five different mice (n = 5, *p<0.001 compared to sham or MI group, student's t-test). The one way ANOVA test among the groups of MI and MI+Tβ4 also showed significant at p<0.05 level. B. Immuno-histological analysis was performed by Masson's trichome staining in MI and Tβ4 treated MI mice.

To find out the role of Tβ4 in cardiac fibrosis, we analyzed collagen type I and type III mRNA expression in 7d post-MI mice. Our data indicate that collagen type I (52.35±1.6 *vs.* 123.82±2.15, p<0.001) and type III (61.36±1.7 *vs.* 185±2.1, p<0.001) were significantly upregulated in 7d post-MI mice. Furthermore, Tβ4 significantly reduces both collagen I (123.82±2.15 *vs.* 67.05±1.37, p<0.001) and collagen III (185.2±2.1 *vs.* 86.03±1.5, p<0.001) expression compared to non-treated MI mice. (Fig. 6A). Additionally, Masson's trichome staining showed a significant reduction in interstitial cardiac fibrosis in Tβ4 treated MI mice (Fig. 6B)

## Discussion

The present study shows for the first time that the PIP complex is associated with human DCM and in experimentally induced heart failure mouse models like MI and TAC. Mechanistically, we demonstrated that treatment with Tβ4 in the MI model results in significant improvement of cardiac function by suppressing NF-κB and collagens synthesis, suggesting a potential use of Tβ4 for therapeutic intervention. LV remodeling is a complex process that involves both infarcted and non-infarcted areas in the post-MI myocardium resulting in LV dilation and ultimately heart failure. In light of ILK's role in cardiac pathologies, recent studies have shown that transgenic mice with cardiac-specific over expression of ILK exhibited compensated cardiac hypertrophy [21] whereas ablation of ILK in mice caused more advanced cardiac failure [8]. These data strongly demonstrate that the ILK level is vital for the control of cardiac function and dysfunction, and supports ILK as a suitable target for therapeutics. The present study shows that ILK is dramatically elevated in human DCM hearts further establishing a potential role of ILK in heart failure. Importantly, we showed that the ILK elevation was accompanied by an upregulation of its partner molecule PINCH-1 and α-Parvin as well. The induction of the PIP complex at this point may be evaluated as a consequence of a stress or injury related phenomena where it acts as a signal to regulate the remodeling process. This correlates with extensive cell biology studies demonstrating a critical role of PINCH-1 in controlling the ILK function in FA [13], [17]. Since α-Parvin/ILK/PINCH-1 is known as a major signaling platform within the integrin-actin network [13], our results suggest a possibility that PIP manipulation may lead to novel targets for heart disease.

Another finding of our study was the hyper-phosphorylation of Akt/PKB in failing human hearts. Such a phenomenon was also previously observed in another set of failing human hearts [22]. Furthermore, in ischemia reperfusion (I/R) and ischemic preconditioning (IPC) settings in a rat model, Hausenloy DJ *et al* showed that IPC protects the heart by phosphorylating the pro-survival kinases Akt at reperfusion [23], [24]. Our results corroborate the recent animal model observations that have shown that ILK-mediated Akt/PKB activation is a key pathway for mediating cardiac dysfunction especially during heart failure and repair [8], [15], [16]. Interestingly, Akt phosphorylation was not enhanced during compensated hypertrophy in human hearts [25], suggesting that the hyper-activation of Akt may be involved at the advanced stages of heart failure like DCM. Akt has been implicated to in receptor mediated signaling for survival or contractility of myocytes [26], [27] On the contrary, it has been reported that the phosphorylation of Akt tends to decline in a canine model of pacing induced heart failure [28]. The report showed that in early pacing, both the Akt phosphorylation and kinase activity were increased but were declined in the late pacing *albeit* both were increased in comparison to sham [28]. The discrepancy is likely due to the differing techniques to induce heart failure. We note that although ILK acts as a pseudo-kinase that may not directly phosphorylate Akt, recent studies have indicated that both PINCH and α-Parvin are involved in regulating Akt signaling [29]. Specifically, PINCH was found to inhibit PP1alpha and promote Akt activation [29] whereas α-Parvin was found to bind to Akt [30] and possibly promote Akt activation by translocating Akt to the plasma membrane [17]. The detailed pathogenesis of elevated ILK and how its pathway to Akt may regulate human DCM remain to be determined.

We have extended our study to the acute MI mouse model as well as to the TAC model. Our data suggest that PIP complex proteins are elevated in TAC mice suggesting their significant role in pressure induced or chronic heart failure. In an MI setting, our data further showed an enhancement of the PIP complex in 7d post-MI compared to non-MI hearts. We focused our study to the MI as the remodeling occurs immediately in this setting, so that we could assess the cardio-protective effect of Tβ4. LV remodeling after MI involves an alteration of cardiac geometry that include functional, cellular and molecular changes [31] that ultimately result in a deterioration of cardiac function. Post-infarction remodeling occurs at both an early (within 72 hours) and a late phase (beyond 72 hours) [32]. Therefore, a remodeling due to injury has also been considered. To ascertain the role of Tβ4 in post-MI, our data show that Tβ4 treatment further potentiates both ILK and PINCH-1 levels but not α-Parvin-1 level and are associated with improved cardiac function. Another study has shown that ILK therapy was able to attenuate LV remodeling and improve cardiac function after MI which partly corroborates with our study in cardiac protection [33]. Tβ4, the major actin sequestering protein in eukaryotic cells, has several potential clinical applications in the repair and remodeling of ulcerated tissues and solid organs following hypoxic injuries, such as MI and stroke [34]. Tβ4 binds monomer actin in a 1∶1 complex and acts as a buffer, preventing polymerization into actin filaments and maintaining a pool of actin monomers for use when filament synthesis is necessary. Therefore, further upregulation of ILK and PINCH-1 in this study may be necessary to protect the myocardium from excess actin polymerization. In addition, in a separate study by Smart N *et al*, showed an induction of epicardial neovascularization by Tβ4 in the heart having the capacity to sustain the myocardium after ischemic damage [35]. It is, therefore, speculated that a long-term effect of Tβ4 after MI would be more effective. We are currently investigating whether Tβ4 has any role in cardiac mass prevention in the TAC model.

To get insight into the mechanism of Tβ4 action in Akt phosphorylation and PIP signaling, mice were treated with the Akt inhibitor wortmannin in the presence of Tβ4. Activation of PI3K/Akt-dependent signaling has been demonstrated to protect cardiac myocytes from I/R injury and to inhibit I/R-induced cardiac myocyte apoptosis [36]. Treatment with wortmannin in addition to Tβ4 decreased the infarct size attenuating effects of Tβ4 resulting in infarct areas comparable to MI only mice. However, wortmannin treatment minimally attenuated the protective effects of Tβ4 on cardiac function in the acute infarct period one week after MI, which is consistent with other studies that have shown inhibition of Akt signal by wortmannin partially preventing the favorable post-MI process in AT1A KO mice, but not in WT mice [21]. This suggests that the beneficial effects of Tβ4 on cardiac function may not be solely dependent on increased Akt activation. Increased Akt activation may be, in part, responsible for the decreased infarct size seen which is directly related to myocyte survival. It is also possible that Tβ4 activating other signaling pathway for cardiac protection like induction of epicardial neovascularization to overcome the ischemic insult [35]. The reason this did not translate into improved function may be because the additional surviving myocytes in the Tβ4 only *vs.* Tβ4+ wortmannin mice are recovering from ischemic injury in the acute post-MI period and do not significantly contribute to overall contractility [42]. Further studies comparing cardiac function in the remote MI time period would help to determine if the decreased infarct size in Tβ4 only treated mice leads to improved function over that of Tβ4+ wortmannin treated mice.

It is also known that MI induces many inflammatory molecules which further accelerate the adverse remodeling process and that this inflammatory process is regulated by NF-κB [37]. Our data showed that 7d post-MI activated NF-κB was significantly inhibited by Tβ4 treatment suggesting that Tβ4 mediated cardiac protection was partly due the inhibition of NF-κB activation. Inhibition of NF-κB by Tβ4 would provide additional support of cardiac protection by reducing the inflammatory response. This is the first report showing that Tβ4 suppresses NF-κB activation in an MI model. NF-κB, regulates various genes involved in inflammatory responses and its activation is important for host defense and repair following insult [38]. It is, therefore, more reasonable to speculate that the suppression of NF-κB activation is a beneficial effect. Previously, we have shown that either knocking down or complete inhibition of NF-κB significantly reduces cardiac hypertrophy in an animal model [38], [39]. It has also been shown that application of Tβ4 accelerates corneal re-epithelialization and modulates corneal cytokine production in corneal alkali injury model [40], [41], [42]. Tβ4 has been shown to inhibit NF-κB activation in TNFα stimulated corneal epithelial cells [43] providing the evidence of anti-inflammatory function. Our data are in agreement with the anti-inflammatory role of Tβ4 after MI. Furthermore, it has been suggested that Tβ4 promotes angiogenesis, which would potentiate its therapeutic benefits in ischemic insult [40], [44].

We also observed reduced cardiac fibrosis post-MI following Tβ4 treatment. This suggests that Tβ4 mitigates cardiac remodeling by decreasing collagen I and III expression and collagen deposition.

In conclusion, our study demonstrates for the first time the complete expression profile of PIP proteins in human DCM hearts, providing a greater understanding of the alteration of cytoskeleton proteins during heart failure. We also show that Tβ4 inhibits NF-κB activity and cardiac fibrosis after MI suggesting a possible mechanistic pathway by which Tβ4 aids in cardiac function and repair in the setting of acute MI. As reported earlier, Tβ4 promotes corneal re-epithelialization, wound healing and cell survival while regulating inflammation after trauma or surgery without any significant side effects. It is of note that Tβ4 has been considered for a phase II clinical trial for acute MI by RegeneRx pharmaceutical (http://www.regenerx.com) and more recently, a randomized placebo controlled study was done in healthy volunteers to examine the dose and toxicity effect. The data show Tβ4 to have no apparent toxicity effect, is well tolerated and is under consideration to be used for acute MI [45]. In light of this and our additional mechanistic insight into Tβ4's beneficial role in cardiac protection, our findings also suggest its therapeutic potential in acute MI.

## Materials and Methods

### Collection of Human Tissues

Non-failing (NF) human hearts were obtained from organ donors whose hearts were not suitable for transplantation but who had no history of cardiac disease. The hearts were from victims of motor vehicle accidents (MVA), gunshot wounds or cerebral vascular (CVA) accidents without any known hemodynamic abnormalities. Failing human hearts (F) were obtained from transplanted patients who had been diagnosed with DCM as described previously [46]. Tissues from F human hearts were collected at the time of transplantation and their clinical parameters have been described in our previous publication [46]. Ventricular tissues were frozen in liquid nitrogen for storage at −80°. For determination of PIP expression, left ventricular tissues were used. All studies involving human tissues were in accordance with institutional guidelines and were approved by research ethics committee as described previously [46]. All human sample data were completed at the Cleveland Clinic. All animal protocols were approved by the institutional review board (IRB) of the Cleveland Clinic and follow up analysis of mice data were performed at Texas A & M was approved by the Institutional Biosafety Committee (IBC) and IACUC.

### Protein extracts and Western blot analysis

F and NF human hearts were washed with cold phosphate buffered saline (PBS) and minced with a sterile blade. Total protein was extracted using T-Per buffer from Pierce as previously described [46]. Samples containing 60 µg of protein were separated on 10% SDS-polyacrylamide gels and were electrophoretically transferred onto PVDF membranes as described previously [46]. The antibodies used in this study including α-Parvin, β-Parvin, and PINCH-1and II were provided by Dr Carry Wu. pAkt, Akt, ILK and PINCH were purchased from Cell Signaling. GAPDH or Actin antibody (Santa Cruz Biotechnology) was used as an internal protein loading control.

### Immunoprecipitation

Cytoplasmic extracts were made from left ventricular tissue from F and NF human samples as described previously [46]. In brief, left ventricular tissue from F and NF human samples were homogenized in ice-cold hypotonic buffer (20 mM 4-(2- Hydroxyethyl)-1-piperazineethanesulfonic acid (HEPES), pH 7.9; 10 mM KCl; 5 mM MgCl2; 0.1 mM EDTA, 0.1 mM ethyleneglycotetraacetic acid (EGTA), 1 mM DTT, 1 mM PMSF, and 300 mM sucrose, with protease and phosphatase inhibitor cocktail). The homogenate was incubated on ice for 20 min with 1% NP-40 and centrifuged. Supernatants containing cytoplasmic proteins were collected and stored at −80°C. Approximately, 500 µg of pre-cleared cytoplasmic protein from each F and NF human hearts was immune-precipitated with 5 µg of α-Parvin antibody for 3 h at 4°C and then 20 µl of protein A/G Agarose was added to each lysate and incubated at 4°C for 16 h. The precipitate was collected by centrifugation at 5,000 rpm for 5 min at 4°C. The pellets from each F and NF sample were washed three times in lysis buffer (10 mM HEPES, pH 7.4, 250 mM NaCl, 1% NP-40, 1 mM PMSF, and protease cocktail), and finally with Laemmli buffer. Equal amounts of samples were loaded into 10% SDS-PAGE gel and western blotting was performed by using ILK and PINCH-1 antibody as a probe as described previously [46].

### Electrophoretic mobility shift assay (EMSA)

EMSA was performed using a double-stranded NF-κB binding site oligonucleotide as a probe as described previously [47].

### Myocardial Infarction Model

Acute myocardial infarction in C57BL6 mice was induced by ligation of the left anterior descending artery (LAD) at 8 weeks of age. Mice were anesthetized with intra-peritoneal injections of a xylazine (5 mg/kg) and, ketamine (80 mg/kg) mixture, placed in a supine position, and ventilated with a respiratory rate of 105 breaths/minute and a tidal volume of 0.25 mL at 60% oxygen. Sternums were shaved, sterilized, and opened under a dissecting microscope (Leica Model M500). Animals were intubated and sternotomy was performed, and the proximal LAD was identified with the use of a surgical microscope (Leica M500) after retraction of the left atrium and ligated with 7-0 Prolene. Blanching and dysfunction of the anterior wall verified LAD ligation. Animals were divided into two groups: one that received an intra-cardiac injection of Tβ4 (500 ng/15 µl PBS) just after the ligation and the control group that received PBS. Mice also received Tβ4 (200 µg/300 µl PBS) or 300 µl PBS intraperitoneally. Intraperitoneal injections were given every day for 3 days following MI and then tissues were collected for experimental use.

To examine the effect of Akt inhibition on Tβ4 treatment, mice were injected with the Akt inhibitor wortmannin (17 µg/kg body weight) concurrently with the Tβ4. Tissues were collected 1 week post-MI for experimental use. Mice received Tβ (200 µg/300 µl PBS) or 300 µl PBS intraperitoneally. Intraperitoneal injections were given the day of surgery, the following 2 days, and then days 5 and 7 following MI. Cardiac function was measured by echocardiography before MI and 7 days post. The dose of wortmannin was chosen following the protocol of Li Y *et al* and Gao J *et al* [48], [49].

### Myocardial Infarct Size Measurement

One week after LAD ligation, mice were euthanized. The hearts were flushed and fixed in 10% neutral buffered formalin. Fixed hearts were embedded in paraffin and serially cut at 5 µm from the apex to the level just below the coronary artery ligation. Alternating sections were stained with Masson's Trichrome. Whole sections were imaged with the Pathscan Enabler IV Slide Scanner (Meyer Instruments, Houston, TX). Infarct size was determined by dividing the epicardial infarct length by the epicardial left ventricle circumference.

### Transaortic Constriction (TAC) model

Mice were anesthetized, intubated and placed on a ventilator as stated above. A sternotomy was performed, and the transverse aorta between the right sub-clavian and the left common carotid arteries was isolated. A single 7-0 suture was placed around the transverse aorta and tightened against a 27gauge needle placed externally on top of the artery to provide a form. After tightening, the needle was removed leaving the artery approximately 75% occluded. Once banded, the sternum was closed using 6-0 silk suture, and the skin was closed using 6-0 prolene suture. Any residual pneumothorax was reduced using a 1 cc syringe with a 20G needle prior to closing the skin. The mice were then ventilated for another 20 to 30 minutes, extubated, and then placed in a prone position on fresh bedding. Sterility was maintained throughout the procedure.

### Determination of cardiac function data collection and analysis

Echocardiography was performed using a Vivid 7 echocardiography machine (GE Medical, Milwaukee, WI). LV mass and velocity of the posterior wall were calculated by bullet equation and tissue Doppler echocardiography (TDE) mode as described previously [39].

### Statistical Analysis

Data are expressed as mean (± S.E). Differences between experimental groups were evaluated for statistical significance using Student's *t* test. Differences with a value of *p*<0.001 were considered significant in failing human hearts compared to non-failing human heart. The values for cardiac function parameters and infarct size were expressed as the mean ± standard error of mean (SEM). The statistical significance between Tβ4 and wortmannin treatment groups and those of the control group were calculated by one-way ANOVA followed by Bonferroni's correction for multiple comparisons using PRISM 5 (GraphPad, San Diego, CA). A p<0.05 were considered significant.
